# Study protocol for the implementation of the Gabby Preconception Care System - an evidence-based, health information technology intervention for Black and African American women

**DOI:** 10.1186/s12913-020-05726-0

**Published:** 2020-09-21

**Authors:** Angela Wangari Walter, Clevanne Julce, Nireesha Sidduri, Leanne Yinusa-Nyahkoon, Jessica Howard, Matthew Reichert, Timothy Bickmore, Brian W. Jack

**Affiliations:** 1 Department of Public Health, Zuckerberg College of Health Sciences, University of Massachusetts Lowell, 61 Wilder Street, O’Leary Library, Suite 540-M, Lowell, MA 01854 USA; 2 Department of Family Medicine, Boston University School of Medicine and Boston Medical Center, 1 BMC Place, Boston, MA 02118 USA; 3 College of Health and Rehabilitation Sciences: Sargent College, Boston University, 635 Commonwealth Avenue, Boston, MA 02215 USA; 4 Department of Government, Harvard University, 86 Brattle Street, Cambridge, MA 02138 USA; 5 Khoury College of Computer Sciences, Northeastern University, 360 Huntington Avenue, Boston, MA 02115 USA; 6 Boston University Institute for Health Systems Innovation and Policy, 180 Riverway, Boston, MA 02215 USA

**Keywords:** Black and African American women, Community Health centers, Health information technology, Healthy Start program, Implementation, Preconception care, Preconception Health

## Abstract

**Background:**

Improving the health of women before pregnancy and throughout a woman’s lifespan could mitigate disparities and improve the health and wellbeing of women, infants and children. The preconception period is important for reducing health risks associated with poor maternal, perinatal and neonatal outcomes, and eliminating racial and ethnic disparities in maternal and child health. Low cost health information technology interventions provided in community-based settings have the potential to reach and reduce disparities in health outcomes for socially disadvantaged, underserved and health disparity populations. These interventions are particularly important for Black and African American women who have a disproportionate burden of pregnancy-related complications and infant mortality rates compared to any other racial and ethnic group in the U.S.

**Methods:**

This is a hybrid type II implementation-effectiveness cohort study aimed at evaluating appropriateness, acceptability and feasibility implementation outcomes, while also systematically examining the clinical effectiveness of a preconception care (PCC) intervention, the Gabby System, for Black and African American women receiving health services in community-based sites. The intervention will be implemented in six Community Health Centers and six Healthy Start programs across the U.S. Each study site will recruit and enroll 25–50 young Black and African American women who will participate in the intervention for a 6-month period. Appropriateness, acceptability and feasibility of implementing the PCC intervention will be assessed using: 1) Qualitative data derived from individual interviews with Gabby System end-users (clients and patients) and site staff; and, 2) Quantitative data from staff surveys, Gabby System usage and uptake. Aggregate health risk and utilization measures collected directly from the Gabby server will be used to examine the effectiveness of the Gabby System on self-reported behavior change.

**Discussion:**

This study will examine implementation outcomes and clinical effectiveness of an evidence-based PCC intervention for Black and African American women receiving services in Healthy Start programs and Community Health Centers. Contextual factors that influence uptake and appropriate implementation strategies will be identified to inform future scalability of the intervention.

**Trial registration:**

ClinicalTrials.gov NCT04514224.

**Date of registration:** August 14, 2020. Retrospectively Registered.

## Background

There are significant racial and ethnic disparities in maternal and child health in the U.S. [1–3] Despite advances in medical technology, Black and African American women across social and economic strata experience disproportionate rates of pregnancy-related complications and infant mortality. During 2011–2016, the maternal death rate for non-Hispanic Black and African American women was 42.4 deaths per 100,000 live births compared to 13.0 deaths per 100,000 live births for white non-Hispanic women [4]. When compared to all other racial and ethnic groups, non-Hispanic Black and African American mothers experience the highest rates of infant mortality (10.97 infant deaths per 1000 live births), preterm birth (delivery < 37 weeks of gestation), and low birth weight, with the latter two being leading causes of infant death [5]. Preconception health and health care (PHHC) for women of reproductive age are critical for reducing the risk of adverse maternal, perinatal and neonatal outcomes, and eliminating racial and ethnic disparities [6]. Evidence-based preconception care (PCC) interventions aim to identify and treat health conditions, assist with behavior modification, and mitigate risk factors that may contribute to poor maternal and infant outcomes [7]. However, despite recommendations from the Centers for Disease Control and Prevention (CDC), [6] and the development of indicators for PHHC, [8, 9] the delivery of PCC- particularly preconception counseling for women of child bearing-age by clinicians in community-based settings remains a challenge [10].

The slow uptake of PCC in healthcare and the lack of engagement between women and healthcare providers in shared decision making about PHHC can be attributed to limited resources (lack of time in the clinical encounter, guidelines, and reimbursement); provider barriers (lack of knowledge of PHHC, lack of clarity on specialty responsible for providing PHHC, poor coordination and organization of PHHC); and patient level factors (lack of knowledge of PHHC, not meeting with a healthcare provider during the preconception stage) [10–14]. Incorporating high quality PHHC with a focus on screening, counseling, health behavior modification and treatment in health care settings, and facilitating patient-provider shared decision making in the clinical process has the potential for reducing disparities in maternal and infant outcomes. Thus far, PHHC implementation research efforts in clinical settings have been limited by small sample size and single-site recruitment, [10] and a lack of frameworks (theoretical or implementation) to support implementation and effectiveness outcomes. Additionally, there is a gap in research documenting implementation of evidence-based PCC screening and education tools specifically designed for Black and African American women in community-based settings.

While the terms Black and African American are often used interchangeably in the literature, in this study, we recognize the heterogeneity of Black and African American racial identities and heritage. Black racial identity refers to having origins of any of the Black racial groups of Africa including the Caribbean, and South and Latin America, while African American identity refers to Americans of African descent with North America ancestry [15–17]. We also recognize that individuals may choose to identify as either or both or a different identify altogether. Within this context, this study is among the first to examine the implementation of an innovative health IT system designed to deliver an evidence-based PCC intervention focused on screening and health promotion [18, 19] in Healthy Start programs and community health centers (CHCs) for Black and African American women in the U.S. Contributions to the literature include: 1) implementation and translational research that is relevant to diffusing evidence-based interventions in community-based settings and, 2) effective interventions designed to curtail disparities in maternal and infant health for this population.

### The Gabby System: an evidence-based preconception care intervention

Health information technology (HIT) is an important tool for information sharing, enhancing consumer engagement, and facilitating shared decision making to improve the quality and efficiency of care [20, 21]. HIT can be leveraged to develop culturally tailored, low cost, and convenient interventions to better reach and reduce disparities in health outcomes for socially disadvantaged, underserved and health disparity populations [22]. Technology-based interventions (e.g. mHealth, eHealth) can be leveraged to enhance patient engagement, support decision making, and improve patient-provider communication. The Gabby System, here forth Gabby, is a HIT intervention programmed to screen for PCC-related risk factors and identify health and social care needs across several domains including: genetic health history; immunizations and vaccinations; sexual and reproductive health; infectious disease; health conditions and medication use; alcohol, tobacco and other drug use; social and emotional well-being; relationships and relational health; healthcare access and affordability; social determinants of health; nutrition and physical activity; and environmental wellness [18]. Gabby is an Embodied Conversational Agent (ECA) that provides comprehensive health promotion and education for each domain, assesses one’s readiness for behavior change, and tailors behavior modification strategies to the user’s risk-specific stages of change [19]. Using conversational speaking style, ECA’s are designed to emulate expert communication skills by focusing on the most salient information, evaluating the patient’s level of understanding, and repeating or elaborating information as necessary [23–25]. ECA’s use speech, gaze, hand gestures, intonation, and other nonverbal modalities to capture the experience of human face-to-face conversation with the end user. Gabby is theoretically grounded in Prochaska and DiClemente’s Transtheoretical Model [26] and uses a variety of health education and motivational strategies to support users’ progression through pre-contemplation, contemplation, preparation, action and maintenance stages of change - thus demonstrating that health behavior change is a process rather than a singular event. Users create an individualized “My Health To-Do List” (MHTDL) that they can work on at their own pace and/or discuss with their healthcare provider. Specifically designed and informed by Black and African American women, [27] the efficacy of Gabby has been demonstrated in two randomized controlled trials, and has successfully identified and resolved health risks for these women [18, 19]. While Gabby has shown strong evidence to support health behavior change in the research setting, the capacity to which these outcomes translate into the daily lives of Black and African American women and in community-based clinical contexts has yet to be determined. Utilizing HIT as an implementation modality has the potential to increase scalability, improve fidelity of information and reduce staff training time.

**Fig. 1:**
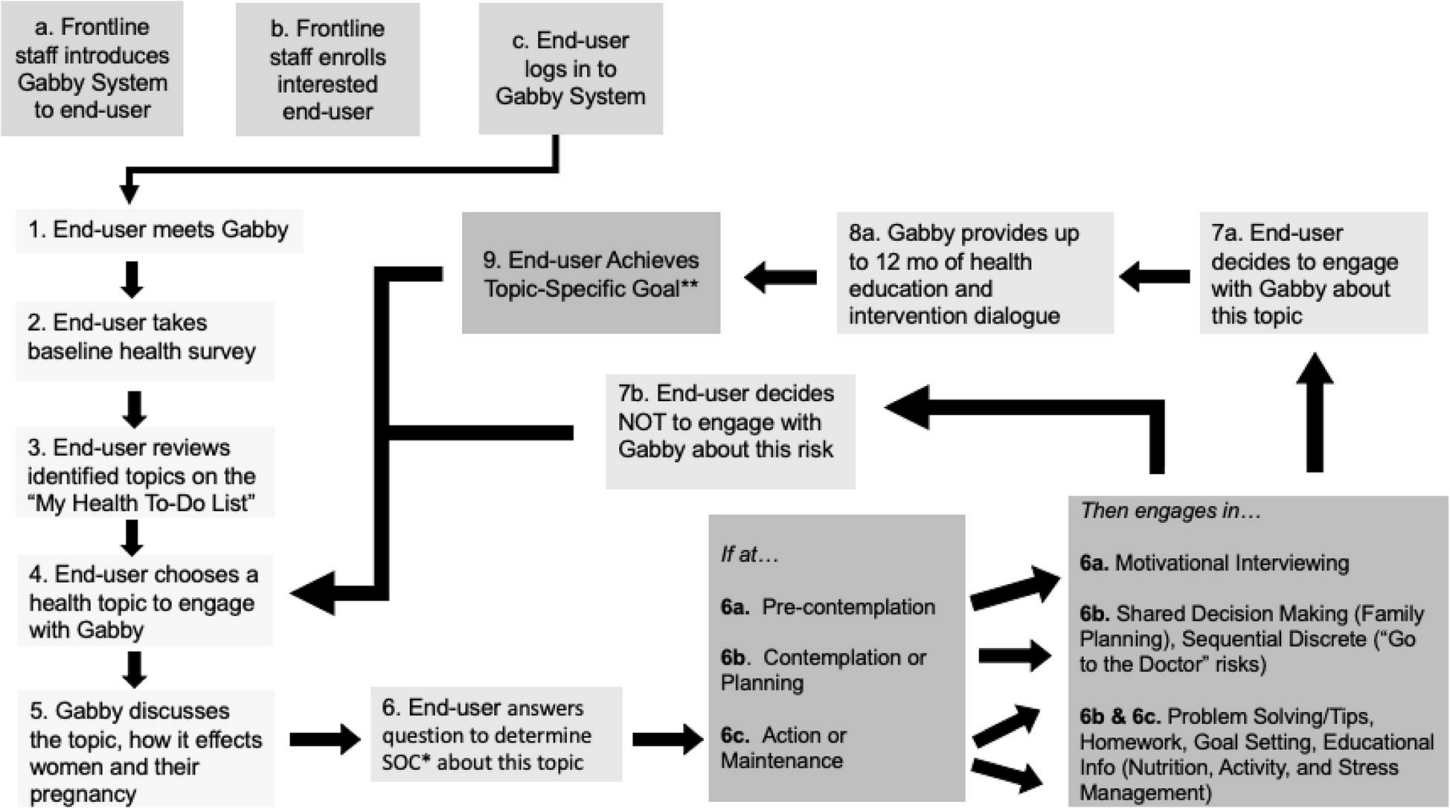
Gabby System interaction from the perspective of an end-user with frontline staff introducing the System

Figure 1 depicts the flow of an initial Gabby System interaction from the perspective of an end-user with frontline staff introducing the system.

### Aims and objectives

The overarching goal of this study is to implement an evidence-based preconception care intervention, Gabby, into community-based health settings. The two co-primary aims will assess the: (1) appropriateness, acceptability, and feasibility of implementing the Gabby intervention in community-based health settings; and (2) effectiveness of the intervention as it is implemented. The aims will be achieved by carrying out the following objectives: (1) engaging six Healthy Start programs and six Community Health Centers as implementation sites and collaborators; (2) assessing clinical workflow and conducting readiness assessments to guide implementation efforts; (3) implementing Gabby at these 12 sites by integrating system use into clinical workflow; (4) assembling a preliminary, revised and final Gabby implementation toolkit; and (5) broadly disseminating the results of this work to community, healthcare delivery and research audiences.

## Methods

We will conduct a hybrid type II implementation-effectiveness cohort study in six Healthy Start programs and six Community Health Centers across the U.S. This study has been reviewed and approved by the Boston University Medical Center Institutional Review Board with the following designations: Not Human Subjects Research for Implementation (H-36816) and Exempt for key informant interviews (H-36716).

### Guiding frameworks

We will use the Proctor and colleagues Implementation Outcomes Framework [28, 29] to guide the evaluation of implementation outcomes (appropriateness, acceptability and feasibility). The contextual factors that influence implementation efforts will be examined and described using the Consolidated Framework for Implementation Research (CFIR) [30]. This determinants framework assembles key domains and constructs across multiple theories, frameworks and models in translational research and represents a comprehensive and flexible model for implementation. CFIR organizes constructs according to descriptive domains - intervention characteristics, outer setting, inner setting, characteristics of individuals, and process. These domains and constructs will shape how we structure our evaluation and describe the nuances of factors that influence feasibility and acceptability of the intervention in community-based health settings. The framework will inform facilitators and barriers to implementation, describe the process of implementation, and identify strategies, modifications and adaptations that sites use to facilitate implementation. The framework will also assist with assessing pragmatic issues of integrating PCC in usual care and identifying appropriate implementation strategies that consider practicalities of inner and outer contexts in which PCC is delivered.

### Settings

Six Healthy Start programs and six Community Health Centers (CHCs) will be recruited to participate in the study. Healthy Start is a national program funded by Health Services Research Administration (HRSA) with a focus on providing education and services to women at high risk for adverse perinatal outcomes before, during, and after pregnancy [31]. Healthy Start programs aim to improve maternal and child health with a focus on reducing the disparities associated with poor birth outcomes [31]. Federally Qualified Health Centers (FQHCs) and Community Health Centers, referred here collectively as CHCs, provide comprehensive health services and are an important point-of-care for low income, socially disadvantaged and rural populations [32]. CHCs are a “one-stop shop” providing an array of primary and preventive care services, including screening, diagnosis, treatment, disease management and social care services to reduce health disparities, morbidity and mortality [32]. Healthy Start programs and CHCs play an important role in serving patient populations that might not otherwise be reached through traditional health delivery systems. Sites will be recruited through several methods that include existing connections made in previous clinical trials, engagement at annual conferences, word of mouth, and strategic emails/calls to sites serving at least 50% African American and Black women.

### Eligibility criteria

The units of analysis for this study represent organizational level participants (clinicians, administrators and staff) and end-users of the Gabby PCC intervention (clients and patients).

#### Organizational level participants

Healthy Start and CHC staff/clinicians/administrators who are employed by the respective organizations are trained in Gabby will participate. Trained staff (community health workers, social workers, clinicians, administrators) will introduce Gabby to the end-users (clients or patients served at the organizations).

#### Clients/patient level participants

Will be female, 18–39 years, self-identify as Black, African American, English speaking, not pregnant at the time of enrollment, have access to internet and receiving services at the Healthy Start or Community Health Center site.

### Sample size and power calculation

To assess implementation based on usage and uptake, we will treat the sample size calculated for effectiveness as a convenience sample. Because usage and uptake are being analyzed descriptively with no baseline data and no over-time comparison, it is not appropriate to calculate power for these outcomes.

To assess effectiveness based on per-end-user stage of change outcomes, our target sample size calculation is based on 12 available sites. Based on our previous RCT of 528 African American/Black women recruited nationally, [19] we expect to see an approximately 7.3% difference in the rate of risks behaviors achieving action or maintenance from 0 to 5.5 months, with a common error variance of approximately 28.7%. With paired two-sided tests, at a power of 0.80 and a significance level of 0.05, we would require at least 125 subjects to detect an effect of at least this magnitude. Because end-users are nested within sites, we also must account for within-site clustering. Based on published intra-class correlation coefficient (ICC) data from 61 clinical practices in 8 practice based research networks on risks such as smoking, diet, and exercise, we anticipate an ICC of 0.012 and a design effect of 1.3 [33]. Based on this design effect, we require at least 160 end-users, or 13 end-users per site. If we anticipate only about 2/3 of end-users to provide outcome data (consistent with our previous study), then we must enroll approximately 240 users, or 20 end-users per site, to detect a change in our outcome from 0 to 5.5 months.

### Implementation strategies

#### Organization-facing implementation activities

**Fig. 2:**
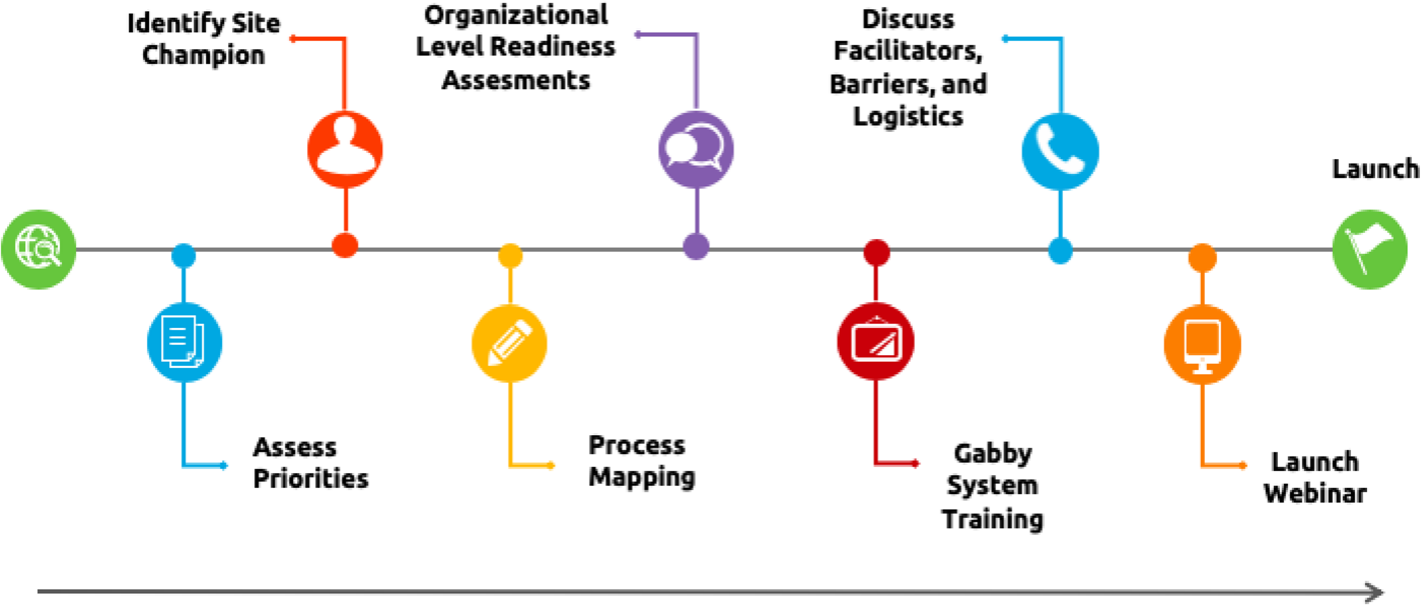
Academic-community partner collaborative Gabby System pre-launch implementation process

As shown in Table 1, we will utilize a set of the Expert Recommendations for Implementing Change (ERIC) strategies [34] at both Healthy Start programs and Community Health Centers. The set of strategies selected account for an understanding of inner and outer contexts that may influence Gabby uptake. The strategies represent activities conducted with community partners in the pre-launch and launch phases of implementation. Prior to launching Gabby, the academic partner (i.e. research staff) will engage participating community partners (i.e. Healthy Start and Community Health Centers) in a collaborative implementation preparation process. Pre-launch activities will consist of: (a) an introductory call; b) an organizational parameters survey; (c) process mapping; (d) an organizational readiness assessment; (e) a one-day site visit; (f) a logistics call with site leadership; and (g) a pre-launch webinar. Figure 2 illustrates the collaborative processes that the community partner will engage in with academic research staff before launching Gabby. Following the launch, technical assistance and relaying clinical data to provider strategies will be used.

**Table 1:** Implementation Strategies by Actor, Action, Target(s) of Action, Temporality & Dosage, Implementation Outcome, and Justification

Strategy	Develop stakeholder relationships	Evaluation and iterative strategies	Train/Educate Stakeholders		Interactive assistance		Use financial strategies
	Identify and prepare site champions	Assess for readiness and identify barriers and facilitators	Distribute educational materials	Conduct educational meetings	Centralize technical assistance	Facilitate relay of clinical data to providers	Alter incentive/allowance structures
ERIC Definition*	Identify and prepare individuals who dedicate themselves to supporting, marketing, and driving through an implementation	Assess various aspects of an organization to determine its degree of readiness to implement, barriers that may impede implementation, and strengths that can be used in the implementation effort	Distribute educational materials (including guidelines, fact sheets, flyers) in person, by mail, and/or electronically	Hold meetings targeted toward different stakeholder groups to teach them about the clinical innovation	Develop and use a centralized system to deliver technical assistance focused on implementation issues	Provide as close to real-time data as possible about key measures of process/outcomes using integrated modes/channels of communication in a way	Work to incentivize the adoption and implementation of the clinical innovation
Actor(s)	Academic partner staff; Community partner leadership, champion, frontline staff	Academic partner staff; Community partner leadership, champion, frontline staff, patients/clients	Academic partner staff; Community partner leadership, champion, frontline staff	Academic partner staff; Community partner leadership, champion, frontline staff	Academic partner staff; Community partner leadership, champion, frontline staff	Academic partner staff; Community partner leadership, champion, frontline staff	Academic partner staff; Community partner leadership, champion, frontline staff
Actions	Introduce Gabby System development history, purpose and duties of site champion, process map rationale. After completion of pre-implementation steps, readiness surveys will be distributed to staff	Organizational readiness and post implementation interviews with leadership, champion and frontline staff; Post implementation focus group with patients/clients	Access to educational and recruitment materials to distribute to staff and patients/clients	Review Gabby System mechanics, content areas, and site-specific implementation approach in an interactive format	Academic partner to address any technical issues identified by community partner in phone calls	Access to site-specific Gabby System administrative page	Monetary incentives, non-monetary incentives such as food or transportation vouchers, Gabby System compatible devices for use at the clinic or during home visits
Target(s) of actions	Community partner leadership (directly); Community partner; champion (directly) Frontline staff (indirectly)	Organizational readiness: Community partner leadership (directly), champion (directly), frontline staff (directly);	Site champion (directly) Frontline staff (directly) Patients/clients (directly) Site leadership (indirectly)	Site champion (directly) Frontline staff (directly) Site leadership (indirectly)	Site champion (directly) Frontline staff (directly) Site leadership (directly)	Site champion (directly) Frontline staff (directly) Site leadership (directly)	Patients/clients (directly) Frontline staff (directly)
		Barriers and facilitators: Patients/clients (directly); Community partner leadership (directly), champion (directly), frontline staff (directly);					
Temporality & Dose	1.5 h completed over 2 months prior to launch	1 h readiness interview prior to launch	Educational and recruitment materials distributed prior to launch	2 h completed during site visit	Weekly calls with community partner staff in Month 1 of launch; As requested by Community Partner Months 2–6 of launch	Ongoing access of administrative page by site staff during Months 1–6 of launch	Use by frontline staff members or patients/clients during implementation
		1 h post implementation informant interview; 1 h patient/client focus group post implementation					
Implementation Outcome	Appropriateness, Acceptability	Appropriateness, Acceptability	Appropriateness, Acceptability	Appropriateness, Acceptability ​​​​​	Acceptability, Feasibility	Feasibility	Feasibility
Justification	Distribution of research information to stakeholders in a readily accessible and succinct manner	Identify and address site-specific weakness in readiness; Make informed decisions regarding allocation of resources and supports; Determine capacity building strategies necessary for scalability	Educational and recruitment materials distributed among community partner staff and patients/clients to aid Gabby System uptake	Provide community partner staff comprehensive overview of intervention features and client enrollment process​​	Provide convenient technical assistance through one-on-one consultations with community partners	Site staff access to administrative page to address and provide follow-up on client risks	Incentive structures support staff implementation efforts and facilitate adoption of intervention by patients/clients

*As defined by ERIC implementation strategies

##### Introductory call (~ 30 minutes)

The primary purpose of this call will be to get buy-in and commitment from the community partner. The academic partner will provide background information about Gabby and convey the purpose and scope of the project to the community partner leadership or administrator(s). The academic partner will collect preliminary data on the organizational infrastructure and population served by the community partner. Community partner sites that commit to the implementation will be asked to identify a site champion who will serve as the primary point of contact to coordinate implementation activities.

##### Organizational parameter survey

The community partner champion(s) or administrator(s) will complete a Qualtrics XM® survey (Additional file 1) providing in-depth information about the organization’s characteristics including organizational partnerships, aggregate funding portfolio, workforce composition and training, staff-client or provider-patient ratios, annual number of end-users, characteristics of the geographic location, and internet and computer accessibility for both staff and end-users.

##### Process mapping

In collaboration with the community champion(s)/administrator(s), the academic partner will create a visual representation of the clinical workflow to get a clear understanding of the workflow processes in the organization. Iterative revisions and clarifications of the process map and a review by community partner leadership and frontline staff will identify any potential roadblocks, areas for improvement within the process, and determine an entry point(s) for the implementation of Gabby to ensure seamless integration into usual care. Process mapping will give insight to the client/patient care structure including individuals involved in the patient/client’s care plan, time allotted for each client/patient, types of services provided in conjunction with preconception care, types of internal and external-facing outreach and engagement activities, types of referrals (from and to whom), levels of case management intensity and location of case management (e.g. home, office visit), and the extent of other data collection and reporting done in the organization.

##### Organizational-level readiness assessment and identification of barriers and facilitators

Using a semi-structured interview guide (Additional file 2) grounded in the CFIR, a qualitative organizational readiness assessment to implement Gabby will be conducted with a diverse group of staff members representing frontline, administrative, and leadership staff at each community site. A quantitative scale based Qualtrics XM® survey (Additional file 3) will be used to concurrently assess organizational readiness and identify barriers and facilitators to implementation.

##### Site visit

The one-day site visit will serve as an opportunity for community and academic partners to interact in person before launching Gabby. The fun, interactive and collaborative site visit will allow for in-depth refinement of implementation activities including: a review of the process map, discussion of readiness assessment findings including how to address identified barriers, and a hands-on training of the Gabby System. At the conclusion of the site visit, key implementation decisions will be refined/finalized to identify persons directly involved in introducing Gabby and enrolling end-users, mechanics of when and how to engage end-users, and avenues for post-launch technical assistance.

##### Site visit follow up logistics call (~ 30 min)

A follow-up conversation will take place after the site visit with the community partner champion and any additional staff members that the champion elects to have on the call. The call will serve as an opportunity to address any concerns or barriers that arose during the site visit, review decisions made during the site visit and iron out details of the Gabby System launch, and make any final changes to recruitment material that will be mailed to the community partner by the academic partner. This discussion is particularly helpful in ensuring that the academic partner and community partner staff are on the same page in regard to implementation details and setting realistic expectations based on the site environment.

##### Launch webinar

A webinar hosted by the academic partner via the Zoom videoconferencing platform [35] will be conducted within 2 weeks of the anticipated start date of each site launching Gabby. During the webinar, the academic partner will review information covered during the site visit including the background and purpose, components of the Gabby System, frequently asked questions, and how to navigate the Gabby System from the end-user and administrator/staff perspectives. The webinar will provide an opportunity to review and clarify major decisions as well as address questions regarding client/patient recruitment and engagement that were discussed during the site visit and subsequent follow-up phone call. As the final step proceeding implementation and following the site visit, the launch webinar will be particularly geared towards frontline staff (e.g. community health workers, case managers) who will be introducing and/or enrolling end-users to ensure that they have the knowledge and tools to carry out implementation tasks.

##### Facilitate relay of clinical data to providers

Community partner staff will have access to the site-specific Gabby administrative page which will provide an overview of the health risks that end-users have identified as issues for concern and discussion. This clinical outcome data will inform providers and staff of health issues that need to be addressed with the client/patient in a follow up visit.

##### Technical assistance

For one month following the launch, the academic partner will provide technical assistance in the form of weekly calls with participating community partners. A semi-structured guide (Additional file 4) will be used to facilitate technical assistance calls and to capture a realistic sense of the progress of recruitment and implementation efforts. The calls will gauge any gaps in the pre-implementation process, resolve issues and identify resources to improve recruitment and engagement of staff and end-users. Technical assistance following the one-month requirement will occur as requested by the community partner. Community partners will also complete monthly implementation logs (Additional file 5) that capture reach (the proportion of Black and African American women willing to participate in the intervention), enrollment (the proportion of women who initiate engagement with Gabby), follow-up and referrals on flagged PCC-related risk factors (e.g. genetic health history, social and emotional well-being, healthcare access and affordability), and any technical issues that end-users and staff encountered. The primary purpose of the implementation log is to provide a quick snapshot of monthly implementation activities that inform reach, enrollment and the nature of interactions that frontline staff have with end-users about Gabby and/or PHHC topics discussed on a monthly basis.

#### Client/patient-facing implementation activities 

##### Engaging consumers: client/patient outreach and engagement

Community partner staff will use multiple means to recruit potential users including introducing Gabby during encounters in the client-staff or patient-provider visit, promoting Gabby at community outreach events, and disseminating Gabby information via the organization’s social media accounts (e.g. Facebook, Instagram, Twitter). Community partners will have an opportunity to follow Gabby on Facebook and Twitter for evidence-based health information and tips, links to health resources, videos, and podcasts specifically designed for Black and African American women, national Maternal and Child Health (MCH) awareness campaigns, and recognition of organizations, celebrities, and leaders who make positive contributions to Black and African American communities. This social media content is aligned with the health topics assessed by Gabby, and therefore will provide an opportunity for frontline staff to discuss health tips and topics that Gabby highlights with their clients or patients.

##### Allowance/incentive structures

A stipend of $1000 has been allotted for each community partner to be used to offset organization-specific challenges in outreach and enrollment efforts (i.e. monetary incentives, non-monetary incentives such as food or transportation vouchers, Gabby System compatible devices for use at the clinic or during home visits). In addition, the academic partner will provide Gabby branded materials including pens, cell phone wallets, tote bags, hand sanitizers, and earbuds to aid in outreach and recruitment efforts.

### Measures and data collection

This hybrid type II cohort study emphasizes both implementation and clinical effectiveness outcomes. We will use mixed methods in the form of individual interviews with staff and end-users, and a quantitative survey of staff to inform appropriateness, acceptability and feasibility outcomes. We conceptualize these mixed methods to be concurrent employing a consistent unit of analysis (staff and organization) across both forms of data, [36] such that qualitative interviews and quantitative survey measures will be collected separately from staff although analyses will complement each other in terms of overall study findings. Both quantitative and qualitative data will assess acceptability, appropriateness, and feasibility. Quantitative client/patient level Gabby System utilization data will be used to assess clinical effectiveness outcomes.

#### Qualitative data collection and measures

The academic partner (i.e. research staff) will engage key stakeholders from each site in virtual interviews prior to launching Gabby. We anticipate that 5–10 participants per site including frontline, administrative, and leadership staff will ensure that diverse perspectives and roles are represented, and will provide sufficient data for saturation, [37] although the exact number will be determined by community partner champions to reflect the organizational structure and workforce of the site. We will develop a semi-structured interview guide based on the CFIR domains (intervention characteristics, inner setting, outer setting, process, characteristics of individuals), select constructs within the domains, [30] and by adapting existing questions from the editable CFIR Interview Guide Tool [38]. Interview questions will examine perceptions of facilitators and challenges to implementing Gabby in community sites, the extent to which Gabby is congruent with the culture of each site, and organizational readiness.

The CFIR framework will also be used to guide identification of determinants of implementation, to select appropriate implementation strategies that match determinants identified, and to understand where those strategies can be employed in the implementation process. Strategy domains such as the action, action target, and implementation outcome affected will be outlined. This will ensure that the process for implementation is tailored to each site, directly informed by staff feedback, involves the engagement of site staff with different roles, and re-examines perceived barriers and facilitators over time. Data from this phase will be used to refine implementation strategies and inform appropriateness and acceptability implementation outcomes.

At the end of the implementation period leaders, clinicians, and administrative staff from each site who participated in the pre-launch phase will be invited to participate in an organizational stakeholder post-implementation virtual interview (Additional file 6). In the event of staff turnover, site champions will identify additional staff members to participate in a post-implementation interview. These data inform the feasibility implementation outcome, including staff perceptions of Gabby, the process of implementing the Gabby System within the community partner’s existing workflow and clinical context, and staff recommendations for scalability.

In collaboration with the community partner, the members of the academic research team will also conduct virtual interviews (Additional file 7) with end-users to evaluate perceptions of usability, usefulness, and cultural relevance of Gabby, and to gain a fuller understanding of the larger clinical context in which the implementation occurred.

With informed consent, all interviews with site staff and end-users will be conducted via the Zoom videoconferencing platform [35] and audio and video recorded. Audio recordings will be transcribed by a professional transcription service and reviewed by a research team member for accuracy. Web conferencing technology is an acceptable method of qualitative data collection especially among geographically dispersed participant populations, [39, 40] and the Zoom platform supports HIPAA compliance.

#### Quantitative data collection and measures

We will quantitatively evaluate implementation outcomes using two proxy measures for implementation: uptake and usage. Uptake will be measured at the end-user level as a binary indicator of whether the end-user logged in to the Gabby System at least once. Usage will be measured also at the end-user level as the number of logins to the Gabby System and the number of minutes spent using Gabby. Usage and uptake will be collected at 3 and 5.5 months.

In addition, following the organizational readiness interview, community partners will be asked to complete a corresponding scale based 33-question Qualtrics XM® survey that will be used to assess organizational readiness while examining affirming, contradictory or conflicting findings elicited during the organizational readiness interview. Participating community partner staff will receive the survey via email from the academic research staff and will be allotted 72 h from the time that they finish their organizational readiness interview to complete the quantitative survey. The purpose of the Likert scale based Qualtrics XM® survey is to complement the organizational readiness interview and serve as a validation of barriers and facilitators identified at the site. Responses along the 5-point Likert scale will consist of Strongly Disagree (1), Disagree (2), Neutral (3), Agree (4), Strongly Agree (5). This approach will allow for rapid data collection and timely turnaround of analysis and stratification of results by type of staff for actionable steps within a relatively short timeframe at every community site during this critical pre-launch phase.

We will evaluate the clinical effectiveness of the intervention through reports produced by the Gabby System server. For each client/patient we will track: (1) baseline stage of change data obtained for each risk identified during the client/patient’s interaction with the Gabby System; (2) follow-up stage of change data to identify the progression (or regression) in the changes associated with each health risk; (3) risk resolution based on the percentage of subjects reaching the action and maintenance stage for each risk at 3 months and 5 ½ months during the implementation period.

### Analysis

#### Qualitative analysis

Two members of the academic research team will jointly code initial interview transcripts as they are completed using evaluation and framework coding methods [41, 42]. Data analysis will be guided by an a priori coding dictionary based on the existing CFIR codebook, definitions, and coding criteria [43]. Data not aligned with the CFIR codebook will be coded using novel codes following concept coding methods [41]. The coding dictionary will be revised iteratively, and coding discrepancies will be resolved by reviewing the coding dictionary and the relevant section of the interview transcript. When revisions to the coding dictionary are finalized and no new codes emerge, both team members will code the remaining interview transcripts independently. Research team members will engage in reflective analytic practices, and codes most frequently used and appearing in at least two interview transcripts will be selected for secondary analysis. A summary of preliminary findings will be presented to the research team during regular team meetings and shared with site staff members during the facilitators, barriers and logistics discussion prior to the Gabby System training (See Fig. 2). Transcript data will be managed using Dedoose version 8.2.14, an online platform that allows for multiple research team members to simultaneously and collaboratively code and analyze transcript data [44].

#### Quantitative analysis

Implementation outcomes (acceptability, appropriateness and feasibility) will be quantitatively assessed based on uptake (whether each end-user logged in to the Gabby System at least once) and usage (number of logins, and number of minutes spent using Gabby, per end-user), at 3 and 5.5 months. By definition, uptake and usage will not have baseline values, but we will descriptively assess uptake and usage from 3 to 5.5 months. We will also use OLS regression with site fixed effects to assess the relationship between uptake or usage and relevant secondary covariates.

Relevant secondary covariates will be assessed using a 33-question Likert scale-based survey. Scale-based responses allow for an interpretable organizational readiness score that can be compared across CFIR domains, constructs, staff levels, and sites. Composite scores will be constructed by calculating the mean score across all survey questions and aggregated into a broader organizational readiness score. Descriptive analysis will be used to assess the relationship between composite scores and site level characteristics. Independent t-tests will also be used to assess the relationship between domain composite scores and site characteristics; for any subgroup analyses or to control for additional covariates, OLS regression will be used. In addition, domain scores and aggregate organizational readiness scores will be assessed at the individual staff level in two ways. First, OLS regression with site fixed effects will be used to assess the relationship between staff-level domain and organizational readiness scores and staff characteristics. Second, a multi-level random-effects model will also be used to assess relationships between staff-level domains and organizational readiness scores and the same staff characteristics. Thematic codes derived from qualitative interview data described above will be used to support and provide context for scale-based responses. This mixed-methods approach to assessing organizational readiness allows for a broader understanding between the relationship of Likert scale responses and inclusive narrative from stakeholder interviews.

To assess the effectiveness of Gabby, we will measure the rate of risk behaviors per subject at action and maintenance. Stage of change data will be collected at the initial assessment, at 3 months, and again at 5.5 months, and then compared across the three data collection points for triggered risks. We anticipate that subjects receiving an effective Gabby intervention should see a greater number of risk behaviors, from baseline to 3 and then 5.5 months, that have reached the action or maintenance stage of change. We will also assess the rate of risk behaviors per subject progressing forward on the stage of change scale. We will test for the effectiveness of Gabby by using a Poisson model to regress our stage of change rate outcomes on data collection time point, with site fixed effects. In addition, for each risk, a logistic regression model will be used to regress a binary measure of whether that risk had either remained at the action or maintenance stage or progressed forward, data collection time point, with site fixed effects.

Finally, we will run analyses to determine whether Gabby was differentially effective conditional on baseline stage of change. This will be done by restricting the data only to risks that were reported at “pre-contemplation” at baseline; then by calculating the rate that remained at “action” or “maintenance” or progressed forward, and finally by regressing that rate on the period of data collection using a Poisson model with site fixed effects. This procedure will be repeated by restricting the data to risks reported at “contemplation” at baseline, and again by restricting risks reported at “preparation” and then “action” stages of change. All statistical tests will be run at alpha level a = 0.05 and performed using the R programming language, version 3.4.3 [45].

### Data management

All data including audio and video recordings, documentation of consent and notes will be stored in secured, HIPAA compliant computers, laptops or paper file storage equipment. Copies of audio recordings will be deleted by the transcription service once transcribed, and all original audio and video recordings will be deleted by the research team at the conclusion of the study. Transcripts will be de-identified and assigned unique site identification numbers so that site-specific themes can be identified, and coding patterns can be compared across sites. All research staff are trained in ethical conduct of human subject’s research.

### Monitoring and dissemination

In partnership with the community collaborators, results of this study will be disseminated at local, national, and international community meetings and professional conferences. Results will also be submitted for publication in peer-reviewed journals. We will adhere to each journal’s authorship criteria to determine authorship eligibility.

## Discussion

Evidence-based PHHC screening and education has the potential to improve maternal and birth outcomes particularly for Black and African American women. This article describes the study protocol to assess the appropriateness, acceptability and feasibility, and the clinical effectiveness of a HIT evidence-based PHHC screening and education intervention specifically designed for Black and African American women [27] in Healthy Start programs and CHCs. The study also assesses the clinical effectiveness of the Gabby System which is important given that the implementation of efficacious PCC interventions is scarce in MCH primary and secondary prevention programming. The hybrid type II cohort study design allows for continued adaptation and modification of the implementation process to meet the unique needs of community-based settings and ensure that lessons learned at each site (Healthy Start or Community Health Center) are incorporated to enhance sustainability at the respective site. Healthy Start programs and CHCs predominantly serve socially and economically under resourced communities and serve as important access points for health care delivery for underserved populations, particularly Black, Indigenous, Latinx, and other communities of color.

The Gabby System is promising in its ability to identify, educate and reduce PCC-health related risks among Black and African American women [18, 19]. For Black and African American women who bear a disproportionate burden of maternal mortality, [4] and the highest rates of infant mortality, preterm birth and low birth weight, [5] Gabby serves as a catalyst for discussions with healthcare providers and has the potential to improve communication between end-users and their provider, a common challenge among underrepresented patient populations [46]. By identifying health risks, sharing evidence-based health information and educating end-users about these risks, and offering recommendations to mitigate each risk, Gabby enables end-users to be knowledgeable about their health risks in advance of a staff-client or patient-provider interaction and to be better informed in their separate and collaborative health decision making. In essence, Gabby empowers end-users to be active participants during conversations with their provider (i.e. improving PCC health information and preparing questions) and promotes a more person-centered care process where Black and African American women are able to engage in a fruitful discussion for optimal health.

Successful implementation of low-cost technology PCC interventions in community-based settings that provide services to Black, Indigenous, Latinx, and other women of color in rural and under resourced communities has the potential for significant public health impact. Implementation of the Gabby System presents an opportunity for a less costly means to provide evidence-based assessment and health promotion for Black and African American women designed to lower health risks associated with poor maternal and infant health. The innovative Gabby System allows professional care providers in Healthy Start programs and CHCs to deliver the PCC intervention across the care continuum with the highest fidelity and quality (i.e. PHHC content is consistent and evolving by incorporating the most up to date information in each health domain) which is valuable for end-users and care providers.

Broadening the delivery of Gabby, a PCC intervention that has proven to be effective, can foster tremendous public health impact particularly by reducing PCC-related health risks and eventually maternal and infant health outcomes for Black and African American women. Demonstrating the feasibility, acceptability and appropriateness of implementing this intervention in community-based settings has the potential to create credibility, enthusiasm and consensus building for scaling up the intervention to Healthy Start programs, CHCs and other community-based settings across the U.S. To promote opportunities for scaling up in community settings, we will document the implementation of the Gabby System into the real-world clinical workflow of Healthy Start programs and CHCs to inform the creation of a comprehensive implementation toolkit for future dissemination efforts. Adaptations and modifications to the implementation process and Gabby intervention will be captured to inform broader implementation and spread. These real-world settings provide the contextual factors (organizational, staff/provider, client/patient) that must be accounted for during the refinement of the intervention and implementation strategies, and scale-up of such interventions. This knowledge will also begin to inform future implementation science research assessing outcome measures related to cost, penetration and sustainability of the Gabby intervention in community-based organizations.

An interactive implementation toolkit will be made available in an online platform on the Gabby website and will include eight evidence informed learning modules consisting of: 1) Welcome and background of the Gabby System development and rationale; 2) How the Gabby System works; 3) How to begin implementation; 4) Outreach and engagement strategies; 5) Monitoring and evaluation; 6) Sustainability and regeneration efforts; 7) Lessons learned; and 8) A compendium of “Frequently Asked Questions” (FAQs) and other useful information that can be accessed by implementation sites and other stakeholders interested in Gabby.

Lastly, this study will demonstrate how academic-community partner collaborations can be leveraged to implement evidence-based interventions in community-based clinical sites within the existing clinical workflow. We will identify best practices to implement HIT preconception care interventions in community-based settings, so our methodology is replicable for widespread implementation in other settings and environments. We will develop an implementation blueprint (with exemplary implementation strategies) that addresses barriers and facilitators to inform and refine implementation in Healthy Start programs and Community Health Centers.

### Study status and special considerations

This study is now in the field and with modifications. As a result of the significant disruption that is being caused by the COVID-19 pandemic, the site visit pre-launch activity will be adapted to a virtual implementation facilitation session. Community partners will make adjustments to outreach and engagement efforts to virtual and other creative formats. Additionally, we anticipate community partners to grapple with changes in funding and staff turnover as a result of the pandemic. As such, community partners will be expanded to include community based primary care clinics as collaborators. Data collection for each community partner will include modifications and adaptations specific to the intervention and implementation before and after COVID-19. Analysis of implementation and clinical effectiveness outcomes will account for these time periods and changes across and within the organization.

## Supplementary information

**Additional file 1.** Organizational parameter survey. Survey used to collect in-depth information about the organization’s characteristics. Survey administered using Qualtrics XM®.

**Additional file 2.** Organizational readiness assessment interview guide. Grounded in the CFIR this tool is used to assess organizational readiness and identify barriers and facilitators to implementation.

**Additional file 3.** Quantitative readiness assessment survey. This readiness assessment is used to concurrently assess organizational readiness and identify barriers and facilitators to implementation. Survey administered using Qualtrics XM®.

**Additional file 4.** Technical assistance semi-structured interview guide. This tool is used to facilitate phone calls with site champions and staff engaged in implementation efforts to capture progress of recruitment and implementation efforts.

**Additional file 5.** Monthly implementation log. This tool is used to assess engagement and enrollment of end-users from the perspective of staff and to capture technical issues that end-users and staff encounter during implementation.

**Additional file 6.** Organizational stakeholder post implementation interview guide. This guide is used to assess leadership, clinical, and administrative staffs’ perceptions of Gabby and the process of implementing the Gabby System as well as recommendations for scalability.

**Additional file 7.** End-user post implementation interview guide. This guide is used to examine end-users’ experiences using Gabby, and better understand the context in which implementation occurred from the end-user perspective.

## Data Availability

Not applicable. Data Sharing: De-identified site-level and individual-level data will be available upon request. Data will be shared with researchers who provide a methodologically sound proposal to achieve aims related to primary outcomes. Proposals should be sent to angela_walter@uml.edu. To gain access, data requestors will need to sign a data access agreement.

## Abbreviations

CDC: Centers for Disease Control and Prevention
CFIR: Consolidated Framework for Implementation Research
CHC: Community Health Center
FQHC: Federally Qualified Health Center
HIT: Health Information Technology
HRSA: Health Resources and Services Administration
MCH: Maternal and Child Health
MHTDL: My Health To-Do List
PCC: Preconception Care
PHHC: Preconception Health and Health Care
