# Supplementary material for: Study protocol for the implementation of the Gabby Preconception Care System - an evidence-based, health information technology intervention for Black and African American women
Source: BMC Health Serv Res. 2020 Sep 21;20:889. doi: 10.1186/s12913-020-05726-0 (PMC7504872; doi:10.1186/s12913-020-05726-0)
Supplement: Supplementary file 1 — Additional file 1. Organizational parameter survey. Survey used to collect in-depth information about the organization’s characteristics. Survey administered using Qualtrics XM®. [file 12913_2020_5726_MOESM1_ESM.docx]

Additional file 1. Organizational parameter survey

The Preconception Care Research Team at Boston Medical Center is implementing the Gabby System across Community Health Centers and Healthy Start sites nation-wide. The purpose of this survey is to assess demographic, clinical, and site characteristics at your location. This survey will take about 10 minutes to complete. Thank you for your time!

Q1 What best describes your site?

- Standalone (operates independently)
- Part of or connected to local public health department
- Part of or connected to a hospital
- Part of or connected to a school
- Other (Specify): ________________________________________________

Q2 Is your site part of the Collaborative Improvement & Innovation Networks (CoIINs)?

- Yes
- No

Q3 For preconception and interconception/postpartum clients, where does care take place? Please describe the percentage (%) of care at each location.

|  | 0-25% | 26-50% | 51-75% | 76-100% |
| --- | --- | --- | --- | --- |
| On-site |  |  |  |  |
| At client's home |  |  |  |  |
| Other (Specify): |  |  |  |  |

Q4 For preconception and interconception/postpartum clients, which type of staff are available for a client to see at your site?

|  | On-site | Off-site | By Referral |
| --- | --- | --- | --- |
| Doctor |  |  |  |
| Nurse/Nurse Practitioner |  |  |  |
| Case Manager |  |  |  |
| Case Worker |  |  |  |
| Administrator |  |  |  |
| Receptionist |  |  |  |
| Registration Staff |  |  |  |
| Community Health Worker |  |  |  |
| Social Worker |  |  |  |
| Outreach Worker |  |  |  |
| Dietitian/Nutritionist |  |  |  |
| Educator |  |  |  |
| Physician Assistant |  |  |  |
| Other (Specify): |  |  |  |
| Other (Specify): |  |  |  |

Q5 Does the same staff member carry out intake and case management?

- Yes
- No

Q6 Who does the intake for preconception and interconception/postpartum clients?

- Nurse/Nurse Practitioner
- Community Health Worker
- Social Worker
- Receptionist/ Administrator/ Registration Staff
- Case Manager/ Case Worker
- Other (Specify): ________________________________________________

Q7 For preconception and interconception/postpartum clients, who primarily provides case management to clients? For the purpose of this question, case management entails intake and follow up processes like assessing, planning, and facilitating services available to meet the client's health needs.

- Case Manager
- Case Worker
- Nurse/Nurse Practitioner
- Physician
- Community Health Worker
- Social Worker
- Other (Specify): ________________________________________________

Q8 Please rate how easy or difficult it is for your site to make referrals to the following services:

|  | Very Easy (1) | Easy (2) | Average (3) | Difficult (4) | Very Difficult (5) | N/A (Referral unavailable at my site) | N/A (Referral not needed at my site) |
| --- | --- | --- | --- | --- | --- | --- | --- |
| Medical (Primary Care, Specialty Care: OB/GYN, Dermatology, Cardiology, Neurology, Gastroenterology, etc.) |  |  |  |  |  |  |  |
| Therapies (Occupational, Physical, Speech Language) |  |  |  |  |  |  |  |
| Behavioral Health |  |  |  |  |  |  |  |
| Lactation Specialist |  |  |  |  |  |  |  |
| Early Intervention |  |  |  |  |  |  |  |
| Social Services |  |  |  |  |  |  |  |
| Child Care |  |  |  |  |  |  |  |
| Domestic Violence |  |  |  |  |  |  |  |
| Education |  |  |  |  |  |  |  |
| Food |  |  |  |  |  |  |  |
| Housing |  |  |  |  |  |  |  |
| Smoking Cessation |  |  |  |  |  |  |  |
| Substance Abuse |  |  |  |  |  |  |  |
| Other (Specify): |  |  |  |  |  |  |  |
| Other (Specify): |  |  |  |  |  |  |  |

Q9 Is there a computer with internet access at your site that clients can use?

- Yes
- No

Q10 What percent (%) of the time is a computer or laptop with internet access available at your site for a client to use?

- 1-10%
- 11-20%
- 21-30%
- 31-40%
- 41-50%
- 50+%

Q11 What percentage (%) of your staff have Community Health Worker (CHW) certifications?

- 0-15%
- 16-30%
- 31-45%
- 46-60%
- 61-75%
- 76%+

Q12 What is the ethnic makeup of the **staff**? (%). Please estimate if you do not have staff self-report data available.

Not of Hispanic, Latinx, or Spanish origin : _______

Mexican, Mexican American, Chicano : _______

Puerto Rican : _______

Cuban : _______

Another Hispanic, Latinx, or Spanish origin. (Specify): : _______

Total : ________

Q13 What is the racial makeup of the **staff**? (%) Please estimate if you do not have staff self-report data available.

White : _______

Black or African American : _______

Asian : _______

Native American or Alaska Native : _______

Native Hawaiian or Other Pacific Islander : _______

Biracial/Multiracial : _______

Other (Specify): : _______

Total : ________

Q14 What is the ethnic makeup of the **clients**in your setting? (%). Please estimate if you do not have client self-report data available.

Not of Hispanic, Latinx, or Spanish origin : _______

Mexican, Mexican American, Chicano : _______

Puerto Rican : _______

Cuban : _______

Another Hispanic, Latinx, or Spanish origin. (Specify): : _______

Total : ________

Q15 What is the racial makeup of the **clients**in your setting? (%). Please estimate if you do not have client self-report data available.

White : _______

Black or African American : _______

Asian : _______

Native American or Alaska Native : _______

Native Hawaiian or Other Pacific Islander : _______

Biracial/Multiracial : _______

Other (Specify): : _______

Total : ________

Q16 What geographic setting is your site located in?

- Urban Area (50,000+ people)
- Urban Cluster (2,500-49,999 people)
- Rural (<2,500 people)

Q17 What is the number of clients seen **annually?**

|  | 0-200 | 201-400 | 401-600 | 601-800 | 801-1,000 | 1,000+ |
| --- | --- | --- | --- | --- | --- | --- |
| Preconception |  |  |  |  |  |  |
| Prenatal |  |  |  |  |  |  |
| Pregnant |  |  |  |  |  |  |
| Interconception |  |  |  |  |  |  |
| Postpartum |  |  |  |  |  |  |
| New Clients |  |  |  |  |  |  |

Q18 Please rank the highest level of education your staff members have attained, from **most to least common.**

______ Less than or some high school

______ High school graduate/GED

______ Vocational/trade school or College associate's degree

______ College graduate degree (BS or BA)

______ Postgraduate degree (MS, MA, PhD, MD, etc.)

______ Other (Specify):

Q19 Do you use an evidence-based training curriculum for case management?

- Yes
- No

Q20 Are client-serving staff members required to completed Healthy Start EPIC training modules?

- Yes
- No

Q21 What is the primary language spoken at your site by clients?

- English
- Spanish
- Other (Specify): ________________________________________________

Q22 How many total clients did you serve in the last calendar year?

________________________________________________________________

Q23 What percentage of clients that you serve are reproductive aged women? (ages 15-49)

- 0-15%
- 16-30%
- 31-45%
- 46-60%
- 61-75%
- 76% +

Q24 Feel to use the space below to add any thoughts, suggestions or comments that you want to add.

________________________________________________________________
