# Supplementary material for: Study protocol for the implementation of the Gabby Preconception Care System - an evidence-based, health information technology intervention for Black and African American women
Source: BMC Health Serv Res. 2020 Sep 21;20:889. doi: 10.1186/s12913-020-05726-0 (PMC7504872; doi:10.1186/s12913-020-05726-0)
Supplement: Supplementary file 2 — Additional file 2. Organizational readiness assessment interview guide. Grounded in the CFIR this tool is used to assess organizational readiness and identify barriers and facilitators to implementation. [file 12913_2020_5726_MOESM2_ESM.docx]

Additional file 2. Organizational readiness assessment interview guide

**Prompt:** *<Insert Name Here>, just to start off…*

1. Tell me a little bit about your role and responsibility at your site.

**Prompt*:*** *Now we want to talk a little bit more about your perception of the culture of both your site and your department. When we say culture in the following questions, we mean the norms, values, and general climate of your organization. In the time that you have worked at your site can you…*

2. Describe the overall culture at your site, including satellite sites and partnerships.

3. What is the culture of your team or department?

**Prompt:** *So now we are going to transition into talking about the Gabby system, first can you tell me…*

4. What do you know about the Gabby system?

5. What has administration told you about the Gabby system?

6. How have your colleagues responded after hearing this message from administration?

**Prompt:** *Based on what you know or have heard about the Gabby system…*

7. How does the Gabby system fit into the services that your site already provides?

8. Tell me one way the Gabby system might be helpful to the clients that you serve.

8a. Tell me one way the Gabby system might be helpful to **you** considering the clients that you serve.

9. Describe three advantages of the Gabby system compared to similar interventions

10. Tell me three disadvantages of the Gabby system compared to similar interventions.

11. In your opinion, how easy or how hard is it to use the Gabby system?

12. What barriers might limit your ability to use the Gabby system with your clients.

**Prompt:** *Now we are going to talk about the implementation itself that will be taking place at your site…*

13. Why is the Gabby system being implemented at your clinical site?

14. Describe how that decision was made.

15. How might the Gabby system help your site overall?

16. Who are all of the staff members at your site will be involved in some capacity implementing the Gabby system?

17. Is there anything motivating you and your colleagues to use the Gabby system?

18. What support and resources do you and others at your site **already have or need** in order to successfully implement the Gabby system?

19. What costs will be incurred to implement the intervention?

20. How will the patients that you serve be informed about the Gabby system?

21. Describe any specific plans or procedures your site has developed to implement the Gabby system.

22. Are there plans to collect any data, or elicit clients’ perspectives, during the time your site implements the Gabby system?

23. How confident are you that you will successfully implement the Gabby system at your site?

**Prompt:** *Earlier we talked a little bit about the culture of your team, department and larger organization…*

24. Does the Gabby system reflect the values of your organization?

25. How might this culture affect the implementation of the Gabby system?

26. Tell me about a new idea, intervention, or policy that was recently adopted at your site or within your organization.

27. Think about the organizational structure of your site and how your team delivers services. Will the Gabby system fit into this current structure?

28. Will the Gabby system impact any funding that your site receives?

**Prompt:** *Now I’m interested to learn more about how you think your clients will respond to the Gabby system at your site…*

29. What do you think your clients will say about the Gabby system?

30. Does the Gabby system mirror the values of the clients that you serve?

31. What may influence whether or not your clients consistently participate in the Gabby intervention?

32. Do you think that the Gabby system will be used by your clients who have the greatest need for services?

33. Will your clients have the computer access and time needed to use the Gabby system?

34. What barriers may challenge your clients’ use of the Gabby system?

**Prompt:** *<Insert Name Here> To wrap up our discussion, I’m interested in hearing your thoughts about the future of Gabby at your site based on your goals, values and beliefs. I am also interested in the future of Gabby at your site based on the site’s goals, priorities and the success of this pilot implementation…*

35. Does the Gabby system match your personal values and beliefs?

36. What goals or priority areas **do you have** for your site for the next year?

37. Tell me about any goals or priority areas **that your site or the organization** has for the next year.

37a. How does the Gabby program fit into these goals and priority areas?

38. Do you think the Gabby system will produce long-term benefits?

39. What resources are in place to support long-term implementation of the Gabby system?

40. Will your site still be using the Gabby system a year from now? Why or why not?

41. How will you determine whether or not the Gabby system has been a success at your site?

42. How might others at your site determine whether or not Gabby has been a success?
