# Supplementary material for: Study protocol for the implementation of the Gabby Preconception Care System - an evidence-based, health information technology intervention for Black and African American women
Source: BMC Health Serv Res. 2020 Sep 21;20:889. doi: 10.1186/s12913-020-05726-0 (PMC7504872; doi:10.1186/s12913-020-05726-0)
Supplement: Supplementary file 3 — Additional file 3. Quantitative readiness assessment survey. This readiness assessment is used to concurrently assess organizational readiness and identify barriers and facilitators to implementation. Survey administered using Qualtrics XM®. [file 12913_2020_5726_MOESM3_ESM.docx]

Additional file 3. Quantitative readiness assessment survey

*In this survey, you will be asked to assess various statements regarding site readiness for the implementation of the Gabby system using a 5-point scale: strongly disagree, disagree, neither agree nor disagree, agree, strongly agree.*

*First, we want to talk about your perception of the culture of both your site and your department. When we say culture in the following questions, we mean: norms, values, and general climate of your organization.*

|  | Strongly disagree | Disagree | Neither agree nor disagree | Agree | Strongly agree |
| --- | --- | --- | --- | --- | --- |
| 1. The culture at my site, including satellite sites and partnerships, will aid in the implementation of the Gabby system. |  |  |  |  |  |
| 2. The culture at my team or department will aid in the implementation of the Gabby system. |  |  |  |  |  |

*So now we are going to transition into talking about the Gabby system.*

|  | Strongly disagree | Disagree | Neither agree nor disagree | Agree | Strongly agree |
| --- | --- | --- | --- | --- | --- |
| 3. I know about the Gabby system. |  |  |  |  |  |
| 4. The administration has informed me a lot about the Gabby system. |  |  |  |  |  |
| 5. My colleagues responded positively after hearing about the Gabby system from administration. |  |  |  |  |  |

*Answer these questions* *based on what you know or have heard about the Gabby system.*

|  | Strongly disagree | Disagree | Neither agree nor disagree | Agree | Strongly agree |
| --- | --- | --- | --- | --- | --- |
| 6. The Gabby system fits into the services my site provides. |  |  |  |  |  |
| 7. The Gabby system is helpful to the clients my site serves. |  |  |  |  |  |
| 8. The Gabby system is advantageous compared to similar interventions. |  |  |  |  |  |
| 9. The Gabby system is disadvantageous compared to similar interventions. |  |  |  |  |  |
| 10. The Gabby system is easy to use. |  |  |  |  |  |
| 11. There are barriers that might limit your ability to use the Gabby system with your clients. |  |  |  |  |  |

*Now we are going to talk about the implementation itself that will be taking place at your site.*

|  | Strongly disagree | Disagree | Neither agree nor disagree | Agree | Strongly agree |
| --- | --- | --- | --- | --- | --- |
| 12. My colleagues and I are motivated to use the Gabby system. |  |  |  |  |  |
| 13. My site already has the support and resources needed to successfully implement the Gabby system. |  |  |  |  |  |
| 14. My site needs support and resources to successfully implement the Gabby system. |  |  |  |  |  |
| 15. Costs will be incurred to implement the Gabby system at your site. |  |  |  |  |  |
| 16. There are specific plans or procedures to implement the Gabby system at my site. |  |  |  |  |  |
| 17. There are plans to collect data, or elicit clients’ perspectives, during the time my site implements the Gabby system. |  |  |  |  |  |
| 18. I am confident that I will successfully implement the Gabby system at my site. |  |  |  |  |  |

*Now we will be talking more about the culture of your team, department and organization.*

|  | Strongly disagree | Disagree | Neither agree nor disagree | Agree | Strongly agree |
| --- | --- | --- | --- | --- | --- |
| 19. The Gabby system reflects the values of my organization. |  |  |  |  |  |
| 20. The culture at my organization will affect the implementation of the Gabby system. |  |  |  |  |  |
| 21. The Gabby system will fit into the current organizational structure at my site. |  |  |  |  |  |
| 22. The Gabby system will impact funding that my site receives. |  |  |  |  |  |

*Now I’m interested to learn more about how you think your clients will respond to the Gabby system at your site.*

|  | Strongly disagree | Disagree | Neither agree nor disagree | Agree | Strongly agree |
| --- | --- | --- | --- | --- | --- |
| 23. The Gabby system will be used by clients who have the greatest need for the services. |  |  |  |  |  |
| 24. Clients will have the computer access needed to use the Gabby system. |  |  |  |  |  |
| 25. Clients will have the time needed to use the Gabby system. |  |  |  |  |  |
| 26. There are barriers that will challenge my clients’ use of the Gabby system. |  |  |  |  |  |

*To wrap up our discussion, I’m interested in hearing your thoughts about the future of Gabby at your site based on your goals, values and beliefs. I am also interested in the future of Gabby at your site based on the site’s goals, priorities and the success of this pilot implementation.*

|  | Strongly disagree | Disagree | Neither agree nor disagree | Agree | Strongly agree |
| --- | --- | --- | --- | --- | --- |
| 27. The Gabby system matches my personal values and beliefs. |  |  |  |  |  |
| 28. The Gabby program fits into the goals and priority areas I have for my site for the next year. |  |  |  |  |  |
| 29. The Gabby program fits into the goals and priority areas my site or larger organization has for the next year. |  |  |  |  |  |
| 30. The Gabby system will produce long-term benefits. |  |  |  |  |  |
| 31. There are resources in place to support the long-term implementation of the Gabby system at my site. |  |  |  |  |  |
| 32. My site will still be using the Gabby system a year from now. |  |  |  |  |  |
| 33. There is a measure to assess the success of the Gabby system at my site. |  |  |  |  |  |

Thank you for taking the time to complete this survey. Your response will aid us in streamlining and improving the implementation process. Do you any other comments or suggestions regarding the implementation of the Gabby system at your site?

________________________________________________________________

________________________________________________________________

________________________________________________________________

________________________________________________________________

________________________________________________________________
