# Supplementary material for: Study protocol for the implementation of the Gabby Preconception Care System - an evidence-based, health information technology intervention for Black and African American women
Source: BMC Health Serv Res. 2020 Sep 21;20:889. doi: 10.1186/s12913-020-05726-0 (PMC7504872; doi:10.1186/s12913-020-05726-0)
Supplement: Supplementary file 4 — Additional file 4. Technical assistance semi-structured interview guide. This tool is used to facilitate phone calls with site champions and staff engaged in implementation efforts to capture progress of recruitment and implementation efforts. [file 12913_2020_5726_MOESM4_ESM.docx]

**Additional file 4. Technical assistance semi-structured interview guide**

1. What kinds of technical issues have staff members or clients (i.e. system crash, lagging) encountered while using the Gabby system?
2. Have any clients been enrolled into the Gabby system?
3. What has been the most challenging aspect of introducing the Gabby system to clients?
4. What have been initial perceptions of using Gabby system from clients? On the other end, what have been initial perceptions of using Gabby system from staff?
5. Have you (site champion and/or staff) accessed the admin page yet? How helpful/not helpful has the admin page been?
6. Are there further resources you feel are necessary to aid the recruitment and implementation processes?
7. On a scale of 1-5, (1 being not confident and 5 being very confident) how confident are you that your site will recruit 25-50 women over the next 5 months to use the system?
8. Have been any opportunities for staff members to discuss feedback about the implementation of Gabby with other staff members?
9. What questions did clients have about the Gabby system that staff members were unable or unsure to answer?
   1. Consider asking site champion: What questions did staff members have that you were unable or unsure to answer?
10. Now that your site has started using Gabby, is there anything you would have wanted to know prior to the roll out of the system?
11. Based on what your site has experienced with the roll out, what would you change about the activities that we conducted to prepare for roll out (i.e. process mapping, stakeholder interviews, site visit)?
12. How has the introduction of the Gabby system impacted your current work flow?
13. Our plan is to schedule more weekly technical calls at an ad hoc basis. What do you think about this? How do you foresee the calls being more useful or helpful in the future?
