# Supplementary material for: Study protocol for the implementation of the Gabby Preconception Care System - an evidence-based, health information technology intervention for Black and African American women
Source: BMC Health Serv Res. 2020 Sep 21;20:889. doi: 10.1186/s12913-020-05726-0 (PMC7504872; doi:10.1186/s12913-020-05726-0)
Supplement: Supplementary file 5 — Additional file 5. Monthly implementation log. This tool is used to assess engagement and enrollment of end-users from the perspective of staff and to capture technical issues that end-users and staff encounter during implementation. [file 12913_2020_5726_MOESM5_ESM.docx]

**Additional file 5. Monthly implementation log**

Staff Name: _____________________________________

| **Date** | **# of People Introduced to Gabby (tally)** |
| --- | --- |
|  |  |
|  |  |
|  |  |
|  |  |
|  |  |
|  |  |
|  |  |
|  |  |
|  |  |
|  |  |
|  |  |
|  |  |
|  |  |
|  |  |
|  |  |
|  |  |
|  |  |
|  |  |
|  |  |
|  |  |
|  |  |
|  |  |
|  |  |
|  |  |
|  |  |
|  |  |
|  |  |
|  |  |
|  |  |
|  |  |
|  |  |

| **Date** | **Gabby User ID** | **Any Gabby system issues? Yes/No.** | **If yes, please describe problem.** | **Health Topics Discussed:**  **Yes/No.** | **Action Taken (i.e. referral mode, education provided)** | **Comments, Notes or Follow-up needed** |
| --- | --- | --- | --- | --- | --- | --- |
|  |  | YES NO |  | YES NO |  |  |
|  |  | YES NO |  | YES NO |  |  |
|  |  | YES NO |  | YES NO |  |  |
|  |  | YES NO |  | YES NO |  |  |
|  |  | YES NO |  | YES NO |  |  |
|  |  | YES NO |  | YES NO |  |  |
