# Supplementary material for: Study protocol for the implementation of the Gabby Preconception Care System - an evidence-based, health information technology intervention for Black and African American women
Source: BMC Health Serv Res. 2020 Sep 21;20:889. doi: 10.1186/s12913-020-05726-0 (PMC7504872; doi:10.1186/s12913-020-05726-0)
Supplement: Supplementary file 6 — Additional file 6. Organizational stakeholder post implementation interview guide. This guide is used to assess leadership, clinical, and administrative staffs’ perceptions of Gabby and the process of implementing the Gabby System as well as recommendations for scalability. [file 12913_2020_5726_MOESM6_ESM.docx]

**Additional file 6. Organizational Stakeholder Post-Implementation Interview Guide**

**General Impressions:** I would like to chat about your perceptions of the Gabby System and your role in the implementation process.

1. Describe your role in implementing the Gabby System at your site.
2. Tell me your overall impression of the Gabby System.
   1. What do you think about Gabby?
3. What were some of the challenges you experienced when using the Gabby System?

**Clients:** Let’s talk about clients who used the Gabby System.

1. Describe the clients who were recruited to use the Gabby System.
2. How did you introduce the Gabby System to clients?
3. What barriers limited your ability to engage clients in using the Gabby System?
4. What have clients who used the system told you about Gabby or their experience using the system?
5. What have you heard from clients who tried to use the system, but were unsuccessful?

**Workflow:** Let’s transition and discuss the implementation of the Gabby System at your site.

1. Think about the health survey that clients complete at the beginning. Is that health survey part of or separate from the assessment work already done at your site?
2. Are there any features of the Gabby System, such as the administrative data the system generates, that supports the work done at your site?
3. Who was involved in implementing the Gabby System at your site?
4. In your opinion how has or hasn’t the Gabby System been implemented according to the implementation plan?
5. Can you describe any strategies used to facilitate the implementation process? Examples may include tailoring recruitment materials and using administrative data to learn about system use.
6. Were there any revisions or changes made to the implementation plan to address barriers, challenges, or mistakes?
7. Did any external resources or individuals affiliated with other organizations help you implement the Gabby System?

**Feedback, Goals, and Outcomes:** I am interested in hearing about the process for obtaining feedback about the implementation process, your site’s goals for implementation, and outcomes.

1. Did your site collect any information from staff or clients during implementation of the Gabby System?
   1. If yes, what types of data was collected and how was it assessed and interpreted?
2. Describe any goal(s) that you or your site set for Gabby System implementation.
   1. Was the goal(s) met, changed, or eliminated during the implementation process?
3. Were there any outcomes you were hoping to see in clients who were enrolled in the Gabby System but did not?
4. What is needed to sustain the use of the Gabby System at your site over time?

**Social Media:**  I would like to end by discussing Gabby on social media.

1. Do you or your site follow Gabby on Facebook or Twitter?
   1. If *no* go to question #21 and then STOP
   2. If *yes*, go to question #22
2. Gabby is on social media. You can follow Gabby on Twitter: @gabby_system or on Facebook: @gabbysystem

Have you discussed any of Gabby’s social media posts with clients?

Have any clients discussed Gabby social media posts with you?

1. Have you or your clients been influenced to participate in any health events as a program partner, sponsor, participant, or volunteer based on a Gabby social media post?
2. Have any of your clients contemplated, initiated, or completed a health behavior change due to content from a Gabby social media post?
