# Supplementary material for: Study protocol for the implementation of the Gabby Preconception Care System - an evidence-based, health information technology intervention for Black and African American women
Source: BMC Health Serv Res. 2020 Sep 21;20:889. doi: 10.1186/s12913-020-05726-0 (PMC7504872; doi:10.1186/s12913-020-05726-0)
Supplement: Supplementary file 7 — Additional file 7. End-user post implementation interview guide. This guide is used to examine end-users’ experiences using Gabby, and better understand the context in which implementation occurred from the end-user perspective. [file 12913_2020_5726_MOESM7_ESM.docx]

**Additional file 7. End-User Post-Implementation Interview Guide**

**General Impressions:** I would like to talk about your perceptions of the Gabby System.

1. Tell me your overall impression of the Gabby System.
   1. What do you think about Gabby?
2. How did your conversation with Gabby compare to a conversation with a health care provider?

**System Features and Use:** Let’s chat about your use of the Gabby System.

1. When or how often did you use the Gabby System?
2. Were there any features that were useful or encouraged you to use the system?
3. What were some of the challenges you experienced when using the Gabby System?
4. Think about the health survey that you completed at the beginning. What do you remember about it?
5. Tell me something new that you learned from Gabby.
6. Is there any information you wanted Gabby to tell you but she didn’t?

**Outcomes:** I am interested in learning how the Gabby System might impact your health.

1. Has Gabby helped you manage your health or change any health behaviors?
2. How could Gabby help you prepare for a visit with health care provider?
3. If you had access to the Gabby System, would you still use it a year from now?

**Social Media:**  I would like to end by discussing Gabby on social media.

1. Do you follow Gabby on Facebook or Twitter?
   1. If *no* go to question #13 and then STOP
   2. If *yes*, go to question #14
2. Gabby is on social media. You can follow Gabby on Twitter: @gabby_system or on Facebook: @gabbysystem

Have you discussed any of Gabby’s social media posts with anyone?

1. Have you been influenced to participate in any health events or initiate or complete a health behavior change due to content from a Gabby social media post?
